# A simple and low-cost setup for part per billion level frequency stabilization and characterization of red He-Ne laser

**DOI:** 10.1016/j.ohx.2023.e00421

**Published:** 2023-04-20

**Authors:** Saurabh Kumar Singh, Avinash Kumar, Pranav R. Shirhatti

**Affiliations:** Tata Institute of Fundamental Research Hyderabad, 36/P Gopanpally, Hyderabad 500046, Telangana, India

**Keywords:** Frequency stabilized lasers, Helium neon laser, Fizeau interferometer, Arduino Uno

## Abstract

This work describes the frequency stabilization of a dual longitudinal mode, red (632.8 nm) He-Ne laser, implemented using an open source low-cost microcontroller (Arduino Uno) and its performance characterization using a simple interferometric method. Our studies demonstrate that frequency stability up to 0.42 MHz (3σ, 17 h) can be achieved using this setup. This simple and low-cost system can serve as an excellent part per billion level frequency reference for high-resolution spectroscopy based applications.

## Graphical abstract

**Figure:**
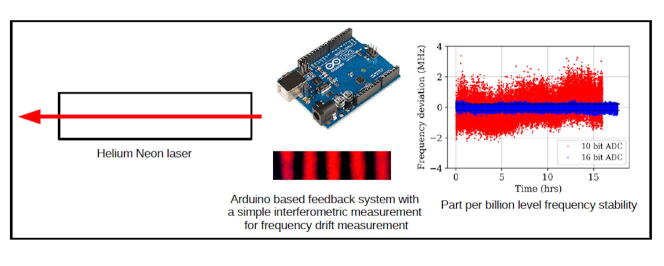

**Specifications table**

**Table:** 

**Hardware name**	*Frequency stabilized Helium Neon (632.8 nm) with a digital feedback control system implemented using an Arduino Uno microcontroller along with diagnostic tools for frequency drift measurement.*	
**Subject area**	• *Physical sciences* • *High-resolution spectroscopy* • *Meteorology*	
**Hardware type**	• *Wavelength reference with part per billion level stability*	
**Closest commercial analog**	*Excelitas Technologies 32734, Newport N-STP-910, Thorlabs HRS015B*	
**Open source license**	*Creative Commons Attribution-ShareAlike 4.0 International license, CC BY-SA 4.0*	
**Cost of hardware**	*Stabilized He-Ne laser approximately: 675 USD and interferometer for frequency drift measurement: 345 USD. Total: 1020 USD*	
**Source file repository**	*https://doi.org/10.5281/zenodo.7152400*	

## 1 Hardware in context

Having a precise and accurate wavelength (or frequency) standard is essential for high resolution spectroscopy related applications. Demands on the level of accuracy and precision of frequency (f), defined as Δf/f, varies over several orders of magnitude depending the application. For example, high-resolution atomic spectroscopy measurements involving ultra-cold atoms/ions often require frequency references with accuracy and precision ranging from $10^{-9}$ to $10^{-15}$ [1]. Typically, lasers locked to well-known atomic/molecular transitions, are used for this purpose.

A common scenario arising in high resolution spectroscopy experiments carried out in internally cold molecular beams, produced by supersonic jet expansion, is that the observed line width is of the order of a few MHz (Δf/f ∼$10^{-9}$) [2]. This is mainly limited by the residual Doppler broadening, caused by a small transverse velocity distribution of the collimated beam, and transit time broadening resulting from a finite interaction time among the molecule and laser beam. Having a simple, low-cost frequency reference with ppb-level stability is immensely valuable in such scenarios. For example, this can serve as a frequency reference in the so-called transfer cavity method for frequency stabilization of lasers operating over a wide range of wavelengths [3], [4], [5], [6], [7]. Here, a frequency stabilized laser acts as a reference for a scanning Fabry Perot cavity, which can then be used to lock another laser operating at a widely different wavelength. Stabilized Helium Neon (He-Ne) lasers are excellent candidates for such applications because of their relatively simple design, good beam quality and long term operational characteristics. However, commercially available systems typically cost greater than 5000 USD, limiting the accessibility of these systems for many applications and situations where lower cost alternatives are desirable.

In this work, we demonstrate an implementation of the well-known polarization stabilization scheme [8], [9], [10], [11] for frequency stabilization of dual longitudinal mode red He-Ne lasers (632.8 nm) using simple and low-cost electronic components. Our design is based on a digital feedback system, implemented on a Arduino Uno microcontroller. This allows for a flexible design and easy visualization of the characteristics to achieve optimal performance. Further, we evaluate its frequency stability by means of a simple interferometric method, built using readily available off the shelf optical components. Using this setup we show that ppb-level frequency stability over several hours timescale, at a fraction of the cost can be attained. This performance is comparable and even exceeds (in some cases) to that obtained by the best of such commercially available systems. We have tested this setup over a time span of more than six months without any noticeable degradation in its performance.

In the following sections, design and implementation of the feedback system for polarization-stabilized He-Ne laser along with the interferometer used for its characterization is described in detail. Following this we discuss the frequency stability achieved for this system using the Arduino controlled feedback system.

## 2 Hardware description

### 2.1 Feedback control system for frequency stabilization

Primary consideration for building such a setup is selecting an appropriate laser cavity that can be used for frequency stabilization by taking advantage of the orthogonally polarized adjacent longitudinal modes [12], [10]. Doppler broadened gain profile of a He-Ne laser spans approximately 1.5 GHz (full width half maximum, FWHM) and using a short cavity with a relatively large free spectral range (FSR) of ∼ 1 GHz, will lead to two orthogonally polarized longitudinal modes within the gain profile. Intensity of these two adjacent modes and corresponding signals (on photodetector) $S_{1}$ and $S_{2}$ at any given instant of time, depend on their position within the gain profile. It will change if there is a frequency drift, for example due to thermal expansion/contraction of cavity. As a result, the normalized intensity difference of the two orthogonal modes can be used to generate an error signal (Δ) for frequency stabilization using the following relation:

(1) $$\Delta =\frac{S_{1}-S_{2}}{S_{1}+S_{2}}$$

**Fig. 1:**
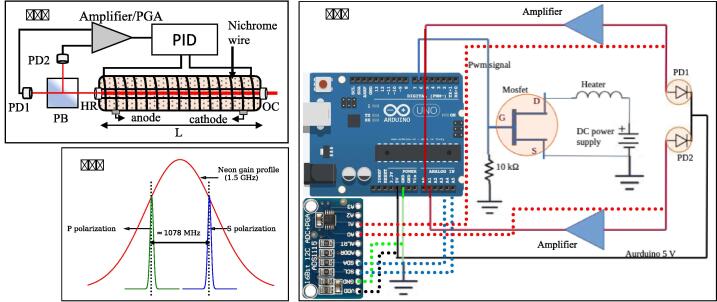
(a) Schematic arrangement of all components used in stabilization scheme. PID corresponds to the proportional-integral-derivative feedback system, PD denotes the photodiode, PB denotes the polarizing beam splitter, PGA denotes the programmable gain amplifier, HR and OC denote the high reflectivity end mirror and the output coupler of the He-Ne tube, respectively. (b) Longitudinal mode structure of He-Ne tube. (c) Schematic representation of microcontroller-based feedback system used for frequency stabilization. In the first approach (solid lines), an analog amplifier and 10-bit microcontroller are used for frequency stabilization whereas, in the second approach (dashed lines), a 16-bit ADC with a PGA has been used.

In this work, we used a He-Ne laser tube with a cavity length of 139 mm (Melles Griot, 05-LHR-006). As per the manufacturer’s datasheet, adjacent longitudinal modes in this tube are spaced by 1078 MHz. Relatively low-intensity light beam emerging from the end mirror (opposite to the output coupler) of the He-He tube was used for frequency stabilization. This beam was passed through a polarizing beam splitter cube (PBS102 – 10 mm, 620 – 1000 nm, Thorlabs) in order to separate the orthogonally polarized longitudinal modes. These two separated modes were made incident on two different photodiodes (FDS100 – Si Photodiode, 350 – 1100 nm, Thorlabs). Photodiode signals (analog) were amplified (5x gain, non-inverting amplifier using OPAmp uA741C IC) resulting in a maximum 4 Volts signal in each channel ($S_{1}$ and $S_{2}$). These signals were digitized using a low-cost microcontroller (Arduino UNO) with a 10-bit resolution (see Fig. 1). A second version using a 16-bit ADC with a programmable gain amplifier (ADS1115) was also built (see SI-1 for details) and tested, resulting in an improved performance.

For the He-Ne tube we used, steady-state temperature under operating conditions was observed to be 60° C (no lock). In order to lock the modes by actively controlling the cavity length, temperature was set a little higher at 65° C. Heating of the tube was achieved by means of a Nichrome wire wrapped around the tube (25 Ω resistance) and applying 12 V across it. Temperature control of the He-Ne tube and hence its cavity length was achieved using a MOSFET (IRF520N) based switching circuit whose duty cycle was controlled by a microcontroller (Arduino Uno). This was also used to provide feedback control by means of the well-known proportional-integral-derivative (PID) technique (source code provided in SI-2).

### 2.2 Fizeau interferometer for frequency drift evaluation

**Fig. 2:**
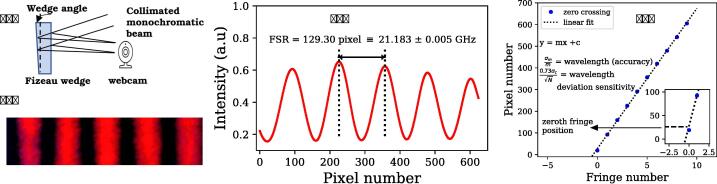
(a) A schematic diagram of the interferometer used in this work for characterizing frequency stable He-Ne (b) Interference fringes captured by a webcam (640 × 480 pixel), using 632.8 nm input (c) Fringe intensity vs. pixel number obtained by summing up the fringe pattern along an axis parallel to the fringes (d) Position of the maxima and minima in terms of pixels vs. the fringe number.

For measuring the frequency drifts of the stabilized He-Ne laser we need a reference that has a precision comparable or better than the He-Ne itself. Some options here are: (1) Mixing the output of our He-Ne laser with an independent stabilized He-Ne laser and monitoring its beat spectrum using a spectrum analyzer (2) Using a high precision interferometer such as commercially available wavemeter or a stabilized high finesse Fabry Perot cavity to monitor frequency drifts. In this work, we chose a simpler alternative that can be implemented with easily available optical components. In principle, the error signal generated by the PID controller itself contains the information about the frequency deviation. However, one needs first to determine the calibration factor using which the change in the error signal can be related to frequency drift. In order to do so, we use a Fizeau interferometer consisting of a 5 mm thick wedge (Thorlabs-WW41050 – Ø1” UVFS Wedged Window, uncoated, ⩽λ/20 over central Ø10 mm) and a low-cost webcam sensor (Quantum QHM495LM6 Webcam, 640 × 480 pixel, lens, and IR filter removed). A schematic diagram of this setup is shown in Fig. 2a. Monochromatic light from the He-Ne laser is coupled through a single mode fiber (Thorlabs - SM, FC/PC, 633–780 nm, FT030-Y, 1 m) and made incident on the wedge. Reflected light from both surfaces forms a characteristic interference pattern (alternating bright and dark fringes) which is recorded using a webcam, as shown in Fig. 2b. A LabView-based program was written to analyze this fringe pattern and obtain a plot of intensity vs. pixel number as shown in Fig. 2c. This is analyzed to obtain positions of the maxima and minima of the intensity pattern. Maxima and minima positions were plotted with fringe numbers and fitted with a straight line to obtain the slope and intercept (see Fig. 2d). The intercept corresponds to the zero fringe position (ZFP) and slope corresponds to the distance between two consecutive crests (or troughs) of the fringe pattern. In essence, uncertainty in wavelength determination is directly related to the error in determining the slope $\frac{\Delta \lambda }{\lambda }=\frac{\Delta m}{m}$. Precision (frequency deviation sensitivity) is related to the uncertainty in ZFP which is defined as $\frac{\sigma_{c}}{\sqrt{N}}$ [13] ($\sigma_{c}$ is uncertainty in intercept determined from fitting, N is the total number of pixels on the horizontal axis). Spacing between two successive crests (or troughs) corresponds to the free spectral range (FSR) of the wedge. A reasonable estimate of the FSR of the wedge can be simply made by measuring its physical thickness as FSR = c/2nL (n = refractive index, L = length). Using L = 4.8 ± 0.1 mm for our wedge and n = 1.45717 for fused silica at 632.8 nm [14], we obtain a value of the FSR to be 21.4 ± 0.4 GHz. A more accurate measurement of FSR, carried using a scanning dye laser calibrated with iodine absorption spectrum, (see SI-3) resulted in a value of 21.1 ± 0.2 GHz. This was cross checked by measuring the change in ZFP for the adjacent modes of the He-Ne spaced by 1078 MHz. A change in ZFP of 6.58 pixels was observed in this case corresponding to the relation: 1 pixel change in ZFP = 163.83 MHz frequency change and FSR = 21.183 ± 0.005 GHz. In summary, independent estimations of the FSR were found to be consistent with each other. Importantly, the relation among change in ZFP with frequency allows us to quantify the frequency drift of the He-Ne laser (see SI-3 for details).

## 3 Design files summary

See Table 1.

**Table 1:** Details of the design used for building the interferometer and feedback control system for stabilizing He-Ne.

Design file name	File type	Open source license	File location
Fig. 1	Schematic figure	zenodo	https://doi.org/10.5281/zenodo.7152400
Feedback control code	Code	zenodo	https://doi.org/10.5281/zenodo.7152400
LabVIEW source code Code	Code	zenodo	https://doi.org/10.5281/zenodo.7152400
CAD file for interferometer base	Onshape stl file	zenodo	https://doi.org/10.5281/zenodo.7152400

## 4 Bill of materials

### 4.1 Details of components used for building stabilized He-Ne laser

See Table 2.

**Table 2:** Details of components used for building the stabilized He-Ne laser.

S. No.	Component name	Part number	Quantity	Approximate cost (USD)	Source
1	He-Ne tube	Melles Griot, 05-LHR-006	1	210	Meredith Instruments
2	He-Ne Laser Power Supply	DG-22–00	1	220	Meredith Instruments
3	Polarizing beam splitter cube	PBS102	1	199	Thorlabs
4	Si Photodiodes	FDS-100	2	22	Thorlabs
5	Dichroic Film Polarizer Sheet	LPVISE2X2	1	9	Thorlabs
6	Microcontroller	Arduino Uno	1	6	
7	OpAmp IC’s	uA741C	2	1	
8	MOSFET switch	IRF520N	4	1	
9	16-Bit ADC	ADS1115	1	4–5	
10	12 W power supply adapter	ECA-12 W-12 12 V 1A	1	2–3	

Total estimated cost:				USD: 675	

### 4.2 Details of components used for the interferometer

See Table 3.

**Table 3:** Details of components used for building the interferometer used for characterizing He-Ne.

Serial number	Component name	Part number	Quantity	Approximate cost (USD)	Source
					
1	Wedge window	WW41050 – Ø1” UVFS Wedged Window	1	105	Thorlabs
2	Single mode fiber	SM, FC/PC, 633–780 nm, FT030-Y	1	74	Thorlabs
3	Fiber coupler	F230FC – B– 633 nm, f = 4.43 mm, NA = 0.56 FC/PC connecter	1	160	Thorlabs
4	Webcam	Quantum QHM495LM6 Webcam, 640 × 480 pixel	1	5	

Total estimated cost				USD: 345	

## 5 Build instructions

### 5.1 Steps involved in building feedback control system for He-Ne laser stabilization

**Fig. 3:**
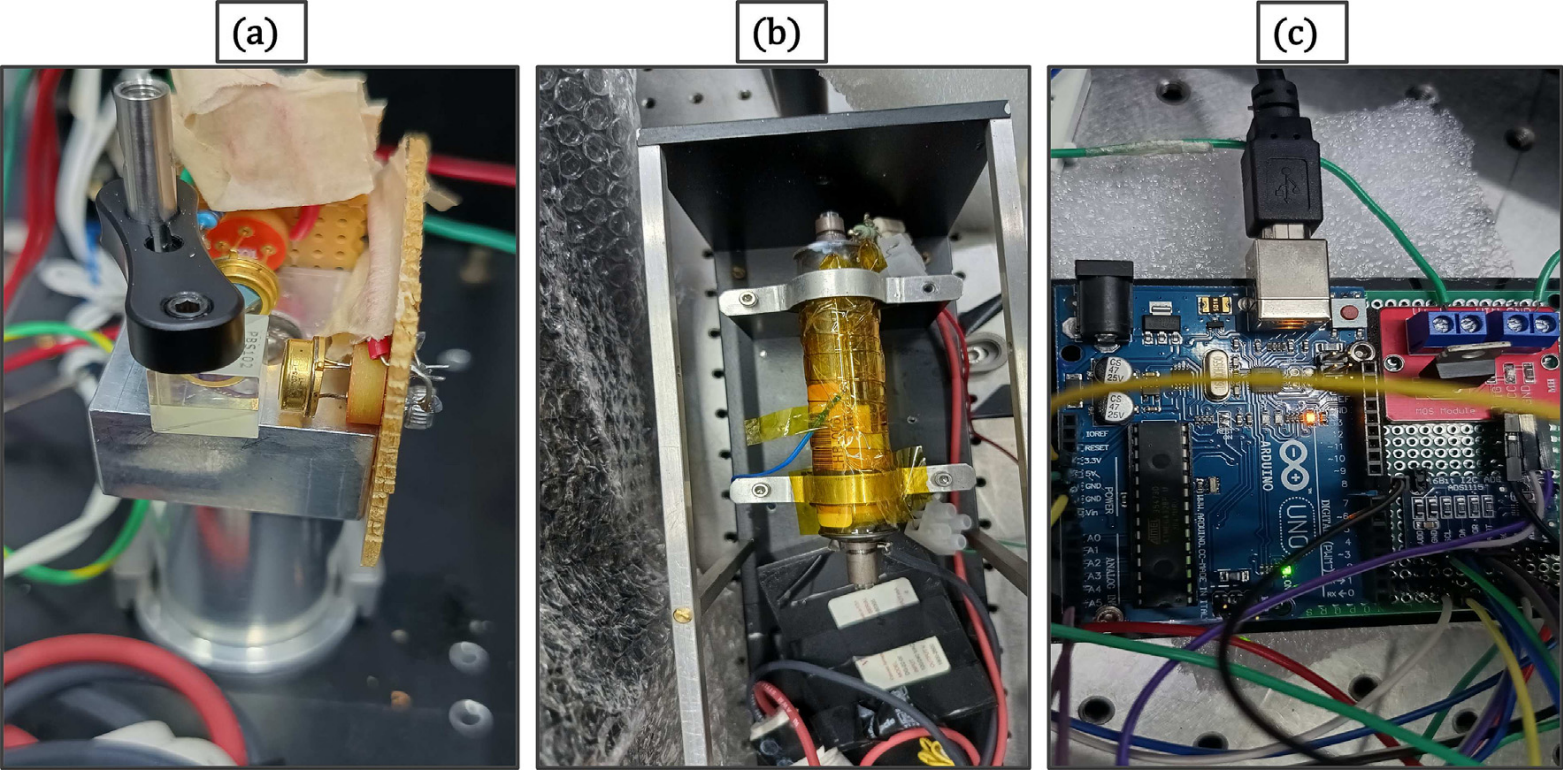
(a) Two photodiodes mounted in a 90 degree orientation with respect to each other around the PBS cube to measure the intensity of the adjacent orthogonally polarized modes (b) He-Ne tube wrapped with nichrome wire, secured with the help of Kapton tape (c) A picture of the digital feedback control system, showing the microcontroller and the MOSFET based switch for stabilization of He-Ne laser.
• Wrap the laser tube with a thin nichrome wire (200-micron diameter) and secure it using Kapton tape (see Fig. 3b). Resistance of the nichrome wire wrapped across He-Ne laser tube was 25 Ohm. With this arrangement, heating time of the laser tube was approximately 6 min, starting from room temperature (25°C) to the locking temperature of 65°C.
• A picture of the feedback control system is shown in Fig. 3c. For reading the analog signals from the photodiodes one can either use the inbuilt 10-bit ADC of the Arduino microcontroller or use an external ADC like ADS1115 with 16-bit resolution for improved precision.
• A schematic diagram of the version using the inbuilt ADC is depicted in Fig. 1c (solid lines), and a version using an external 16-bit ADC (ADS1115) is shown in Fig. 1c (dashed lines). A schematic of the complete circuit used for the 16-bit ADC control system is shown in SI-1.
• The ADS1115 has a built-in programmable gain amplifier (PGA) and with its higher measurement precision provides better locking stability (see validation section).
• We calibrated the error signal (in bits) in terms frequency and the relation was found to be: 1 bit error signal = 0.008 MHz frequency change (see Fig. 8).

• Mount two photodiodes in 90 degree orientation around the PBS cube, as shown in Fig. 3a, and arrange this assembly at the back of the He-Ne tube. These photodiodes measure the leaked light from the highly reflective mirror side of the laser tube. Its magnitude is our case is around 30–40 microWatt. We have used 20 kOhm load resistor (${\text{R}}_{L}$) across the photodiode to amplify the signal (the responsivity of FDS 100 is 0.25 A/W at 633 nm). We observed that the amplified signal was 160 mV, consistent with that estimated from responsivity. (See Table 3)

**Fig. 8:**
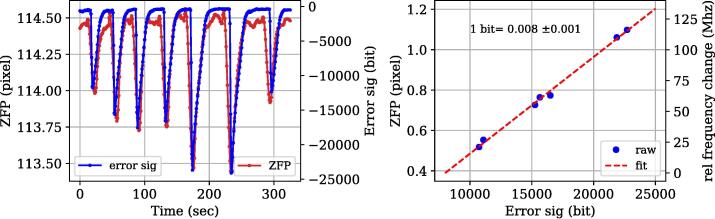
Calibration of error signal (in digital units) in terms of frequency change in MHz. (Left) Changes in ZFP values (red) and error signal (blue) caused by an external disturbance (created by blowing cold air for a few seconds). (Right) A plot of change in the error signal vs. ZFP with a linear fit. Based on the best fit parameters we estimate that change in error signal by 1 bit corresponds to a change in frequency of 0.008 ± 0.001 MHz for the control system with 16-bit ADC. (For interpretation of the references to colour in this figure legend, the reader is referred to the web version of this article.)

### 5.2 Steps involved in building Fizeau interferometer for frequency drift measurement

**Fig. 4:**
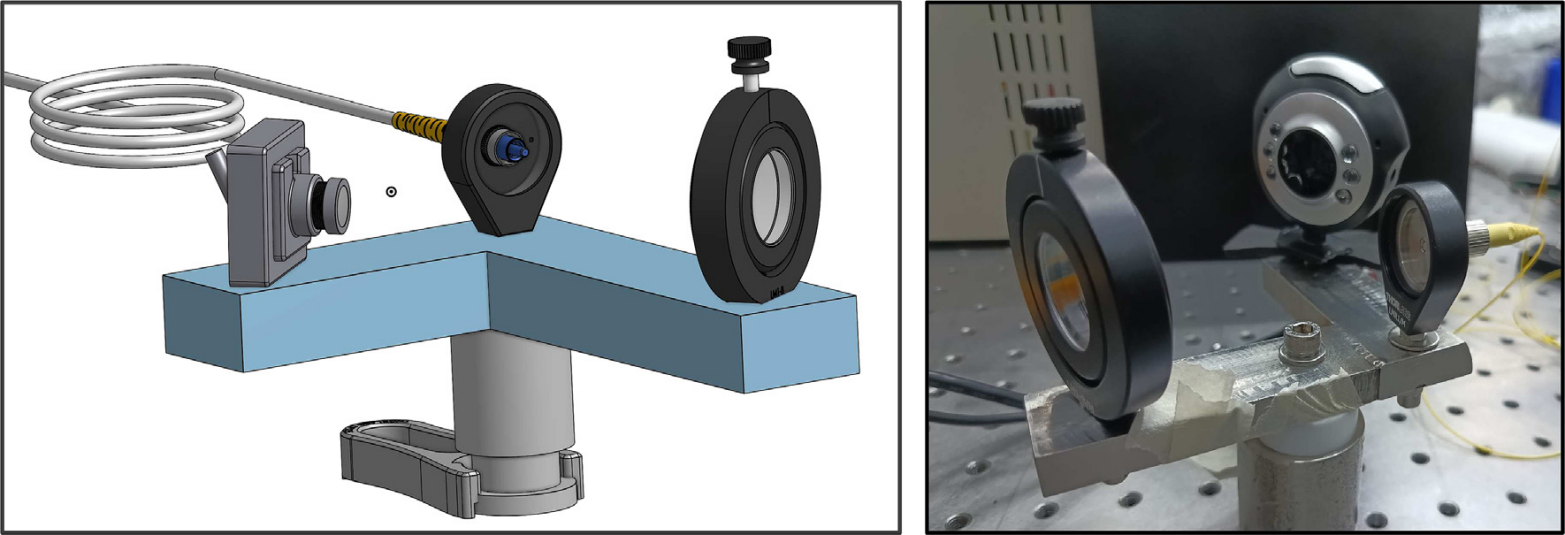
(left) 3D diagram of the interferometer setup (right) picture of interferometer setup that is used for He-Ne laser characterization.
• To improve the thermal stability of the interferometer, we mounted the components on an L-shaped Invar base plate (see design section for CAD file). A Teflon spacer was used to thermally isolate the base plate from other components (see Fig. 4, right).
• Place the webcam sensor such that the interference pattern from the wedged window is captured by a sensor.

• For building the interferometer, take a UV fused silica wedge window and make the He-Ne light output incident on its center using a single mode fiber optic cable (as shown in Fig. 4 (right)).

## 6 Operation instructions

### 6.1 Routine Operation for He-Ne laser stabilization

**Fig. 5:**
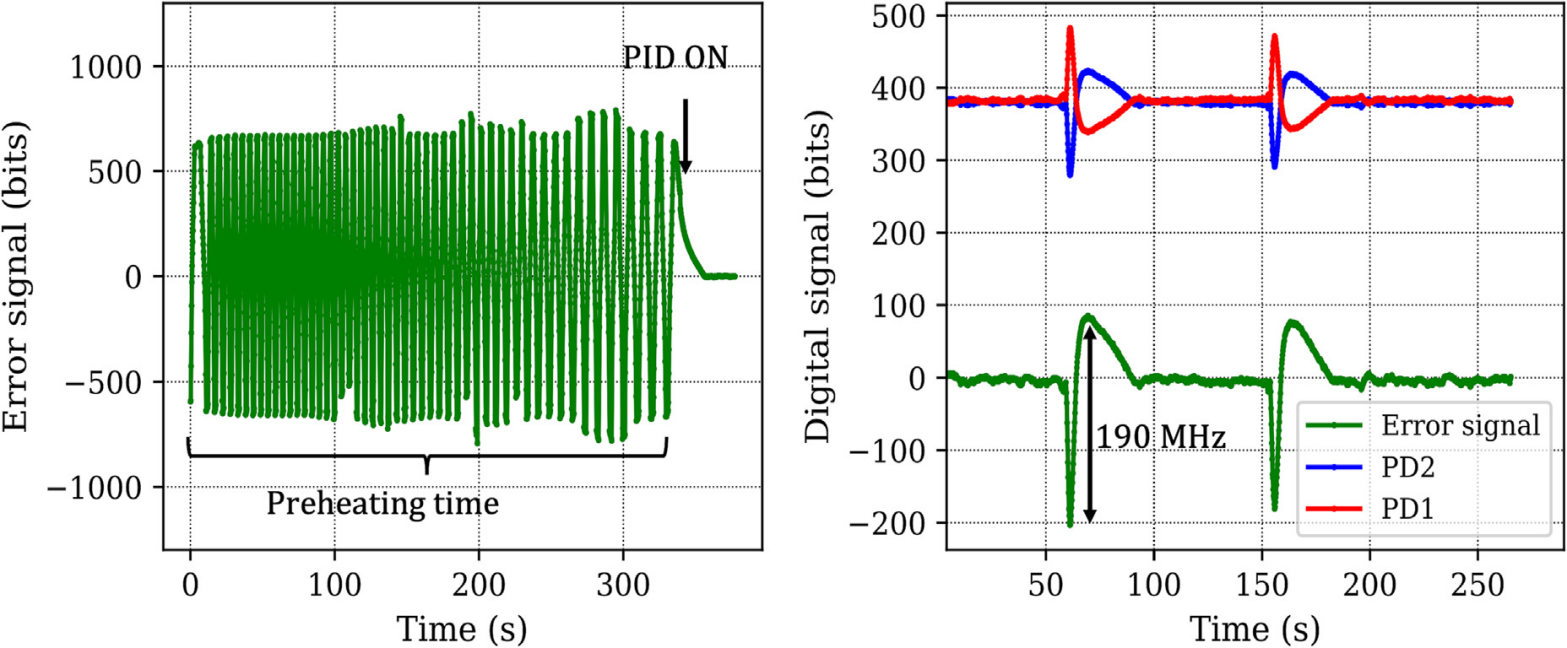
(left) Error signal as a function of time as the preheating is started and the laser reaches stable operation, under active feedback. (right) Response of the stabilized He-Ne to an external disturbance created by blowing cold air on the He-Ne tube directly for a few seconds (around 60 s and 155 s). Our controller is able to lock the He-Ne within 30–50 s.
• To improve the locking stability, decrease the voltage of the power supply gradually by monitoring the error signal. Typically we reduce the heating power to approximately 1 W. At lower powers than this the locking ability gets compromised.

• Turn on the He-Ne laser, power supply for external heater and Arduino microcontroller.
• Upload the sketch code 1 (for preheating) to the microcontroller (see SI-2) for heating the laser tube.
• Monitor temperature of the tube using a sensor (we used a K-type thermocouple for this purpose). Steady state temperature of the tube in our case was 60°C (without external heating). Ensure that the tube is heated to 5–8°C above the steady state temperature with external heating (see SI-2, code 1).
• Once the tube reaches the desired steady state temperature, upload the PID locking sketch code (see SI-2, code 2) in the microcontroller. This control system is very efficient, and it should lock the laser within 30 s (see Fig. 5, left panel). Locking performance can be visualized by looking at the error signal in the plotting interface of the Arduino microcontroller.

### 6.2 Routine operation of the Fizeau interferometer

• Plug any UVC (USB video class) type plug and play webcam to a PC. A LabView interface was used to communicate and acquire data from the webcam to measure ZFP. Source code is provided in the design file section.
• Couple the He-Ne output light into a single mode fiber optic cable and make it incident on the Fizeau wedge.
• Position the fiber optic such that the output beam is incident on the center of the wedge and a good contrast pattern is observed on the webcam (as shown in Fig. 2b).

## 7 Validation and characterization

### 7.1 Locking performance

Typically when our He-Ne laser is turned on (initially at room temperature, 23°C), temperature of the tube increases and reaches a steady state (60°C) in about 15 min, resulting in a passively stable operation. In this duration, the cavity length increases and causes changes in the output frequency, leading to a sweeping behavior of the longitudinal modes, similar to that shown in Fig. 5 (left). Upon actively controlling the cavity temperature by means of external heating, we observed that the time taken to reach a steady state is much smaller and our control system can stabilize the He-Ne laser within ∼ 6 min. Our feedback control system is robust to external disturbances, as seen in Fig. 5 (right panel). Following a disturbance (jet of cold air blown over the He-Ne tube directly) which caused a frequency instability of approximately 190 MHz, the feedback system locks the laser back again in a matter of 30–50 s. Further, by changing the setpoint, it is also possible to scan the output wavelength within the Neon gain profile (see SI-4). Having established that our feedback control system works well, we focus on quantifying the frequency drift under locked condition.

### 7.2 Performance of interferometer

**Fig. 6:**
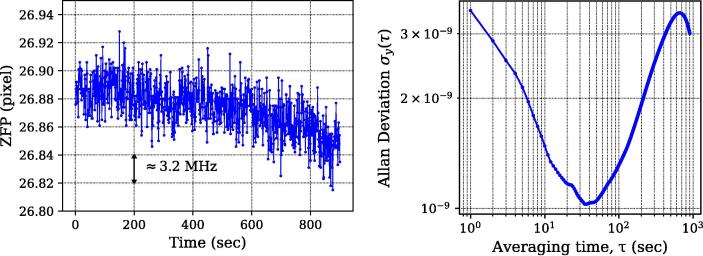
(left) Change in the ZPF values (in pixel) measured for a duration of a few hundred seconds (sampling rate = 30 Hz, with averaging of 30 points, resulting in 1 point per sec). This was measured using the stabilized He-Ne as an input. (Right) Allan deviation corresponding to the data shown in the left panel. In the course of these measurements, the frequency drift in He–Ne was much smaller, as estimated by monitoring the error signal over time. The drift observed correspond to that of the wavemeter itself.

Using the scheme outlined above, we are able to detect changes in ZFP better than 0.02 pixel, ($\frac{\sigma_{c}}{\sqrt{N}}$) resulting in ppb-level precision. It is limited by the thermal drift of the wedge during the course of measurement itself. Using relatively low thermal expansion coefficient materials like fused silica, as in our case, we achieve high precision for a short time (∼15 min, see Fig. 6, left panel). Here, at longer time scales (beyond 400 s), the effect of thermal drift can be seen. Fig. 6 (right) shows the associated Allan deviation. In these measurements the He-Ne laser was locked at a fixed setpoint. Frequency drift of the He-Ne, determined from changes in error signal, was estimated to be much smaller than that observed here. Hence, we conclude that this frequency drift arises largely from the wavemeter itself. A more detailed discussion of the relation between error signal and frequency drift is described in the next section. In principle, this drift can be compensated by measuring the temperature precisely using a high precision (mK) temperature sensor or by housing the wavemeter in a temperature-stabilized housing. However, it should be noted that this short-term stability is good enough to obtain a calibration among the fringe position, error signal and frequency drift. Once this is established, the error signal alone can be used to measure the frequency drift, independently of the long term stability of the interferometer itself.

### 7.3 Relation among the frequency change and the error signal

Frequency drift of He-Ne laser is related to the error signal through intensity changes in adjacent longitudinal modes, as given by Eq. 1. By knowing the precise relationship between the error signal (measured in digital units by our detection system) and frequency drift (obtained using the ZFP shifts), we estimate the frequency stability of the locked He-Ne laser.

**Fig. 7:**
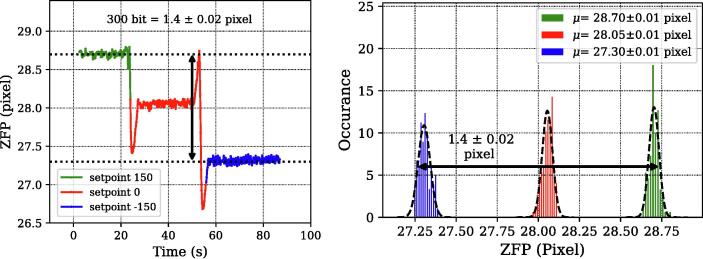
Calibration of the error signal (in digital units) to frequency units (in MHz). (Left) Changes in ZFP values are plotted with different error signal values (setpoint) values. (Right) Histogram of the above measurements showing their distribution and mean values. Based on our calibration (1 pixel = 163.18 MHz) we estimate that 1 bit corresponds to 0.76 MHz. The uncertainty in the mean corresponds to random error and systematic error (thermal drift in interferometer), as described in SI-3. Calibration was done for the 16-bit ADC in a similar manner (see Fig. 8).

In order to do this, ZFP (in pixels) was monitored by changing the setpoint of the PID controller. As we change the setpoint (in digital units), the frequency of the modes under the gain curve shift, and this change was measured using our interferometer. By knowing the previously obtained relation among ZFP shift and the corresponding frequency change (1 pixel = 163.83 MHz), we conclude that one digital unit of error signal corresponds to a frequency change of 0.76 ± 0.01 MHz (see Fig. 7). To increase the readout resolution, we also used a 16-bit ADC with a built-in programmable gain amplifier. To establish a relationship between the error signal and the frequency for the 16-bit control system, we followed a similar strategy. We measured the change in ZFP along with the change in error signal by blowing some cold air directly onto the He-Ne tube. A one-to-one relationship between the ZFP and the error signal could be seen as shown in Fig. 8 (left). Based on these measurements we determined that change in error signal by 1 bit corresponds to a frequency change of 0.008 MHz (see Fig. 8, right).

### 7.4 Long term locking stability

We measured the long-term locking stability of the stabilized He-Ne laser for about 17 h by monitoring fluctuations in the error signal.

**Fig. 9:**
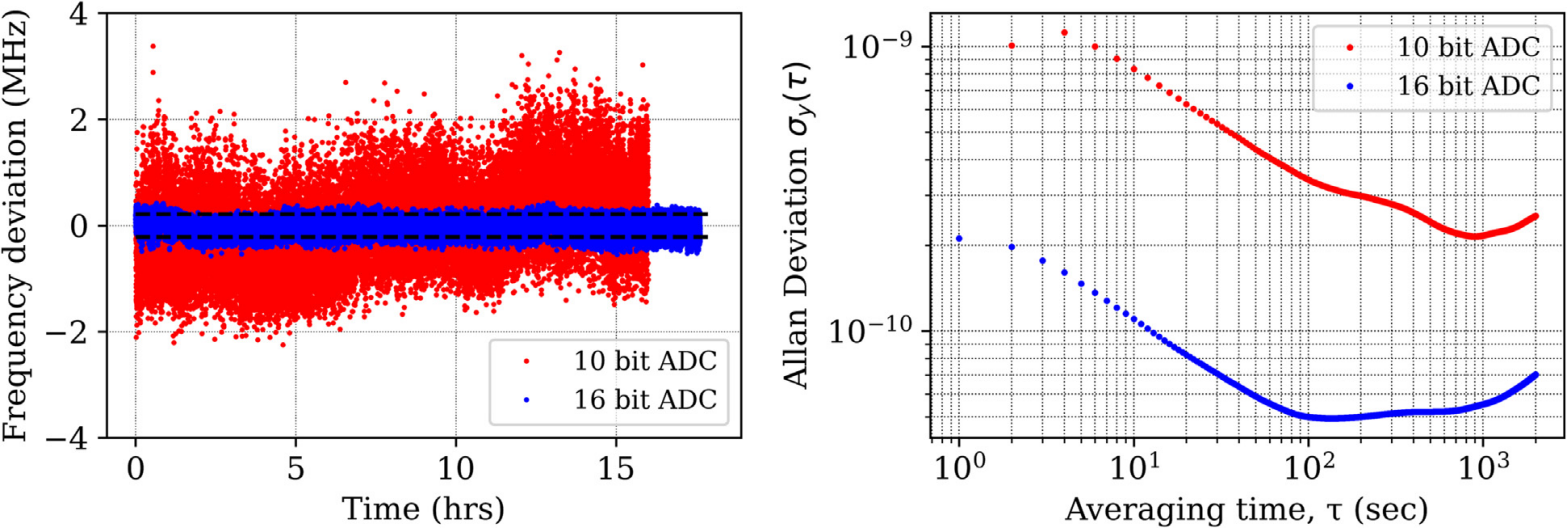
(Top) Frequency deviation in MHz (obtained from change in error signal) measured as a function of time for a duration of 17 h. The frequency stability was found to be 0.42 MHz (3σ) as shown by the two horizontal dashed lines. For the version with 10-bit ADC, it was 3 MHz. (right) Allan deviation plot for the above measurements.

Locking stability (3σ) during this period was observed to be 0.42 MHz (3.0 MHz for 10-bit ADC, 16 h.), as shown in Fig. 9, left). An Allan deviation [15], [16] for these measurements is shown in the right panel of Fig. 9. The stabilized He-Ne system reaches a stability of 5 × $10^{-11}$ for an averaging time of 100 s. In these measurements, our feedback system was measuring the error signal at a rate of 5 Hz. In order to further suppress the noise, 5 such successive measurements were averaged.

In our case, a small amount of back reflection from the fiber input face (P1–630A-FC-1) is enough to decrease the locking stability of the laser (see SI-5). We minimized this by coupling light into the fiber with a slight angular offset such that the back reflection is small. For complete elimination one has to use Faraday isolators or use FC/APC type connectors.

## 8 Concluding remarks

We have successfully demonstrated frequency stabilization and characterization of a He-Ne laser to ppb-level stability, built using simple and low-cost components. Measurement over a time span of 17 h show excellent locking performance with a stability of 0.42 MHz (3σ). Such a device is very valuable frequency reference, especially in applications involving high-resolution laser spectroscopy. Given the simple nature of the design, we also believe that this work has the potential to provide valuable experience in feedback control systems as well as in the learning of fundamental concepts related to lasers when included as part of an undergraduate lab exercise. Finally, this work also provides a preview into the potential of interferometer used in this work. This simple and robust design can be extended to develop a stand-alone high precision and accuracy, low-cost wavemeter. This is currently being pursued in our lab.

## Ethics statements

The work complies with the ethical guidelines of HardwareX and did not involve human subjects or animal experiments.

## **Credit authorship contribution statement**

SKS designed the experiments and performed the measurements and data analysis with inputs from PRS. AK contributed to the experimental design and testing at the initial stages of the work. PRS provided conceptual inputs and designed the project. SKS and PRS prepared the manuscript. All authors discussed the results and contributed to the manuscript.

## Data availability

Files related to the experimental data presented in this manuscript and supplementary information can be accessed from the following link: https://doi.org/10.5281/zenodo.7152400.

## Declaration of Competing Interest

The authors declare that they have no known competing financial interests or personal relationships that could have appeared to influence the work reported in this paper.

## Supplementary data

• SI-1:Circuit diagram o the feedback system used for He-Ne stabilization. • SI-2: PID code for the microcontroller. • SI-3: He-Ne laser cavity and Fizeau wedge characterization. • SI-4: Mode hop free scanning under Neon gain profile. • SI-5: Effect of back reflection on locking stability.
