# Supplementary material for: A simple and low-cost setup for part per billion level frequency stabilization and characterization of red He-Ne laser
Source: HardwareX. 2023 Apr 20;14:e00421. doi: 10.1016/j.ohx.2023.e00421 (PMC10182315; doi:10.1016/j.ohx.2023.e00421)
Supplement: Supplementary file 1 — • SI-1:Circuit diagram o the feedback system used for He-Ne stabilization. • SI-2: PID code for the microcontroller. • SI-3: He-Ne laser cavity and Fizeau wedge characterization. • SI-4: Mode hop free scanning under Neon gain profile. • SI-5: Effect of back reflection on locking stability. [file mmc1.pdf]

A simple and low-cost setup for part per billion level frequency stabilization and characterization of red He-Ne laser – Supplementary Information

Saurabh Kumar Singh, Avinash Kumar, Pranav R. Shirhatti*

Tata Institute of Fundamental Research Hyderabad, 36/P Gopanpally, Hyderabad 500046, Telangana, India
*pranavrs@tifrh.res.in

SI-1: Circuit diagram o the feedback system used for He-Ne stabilization

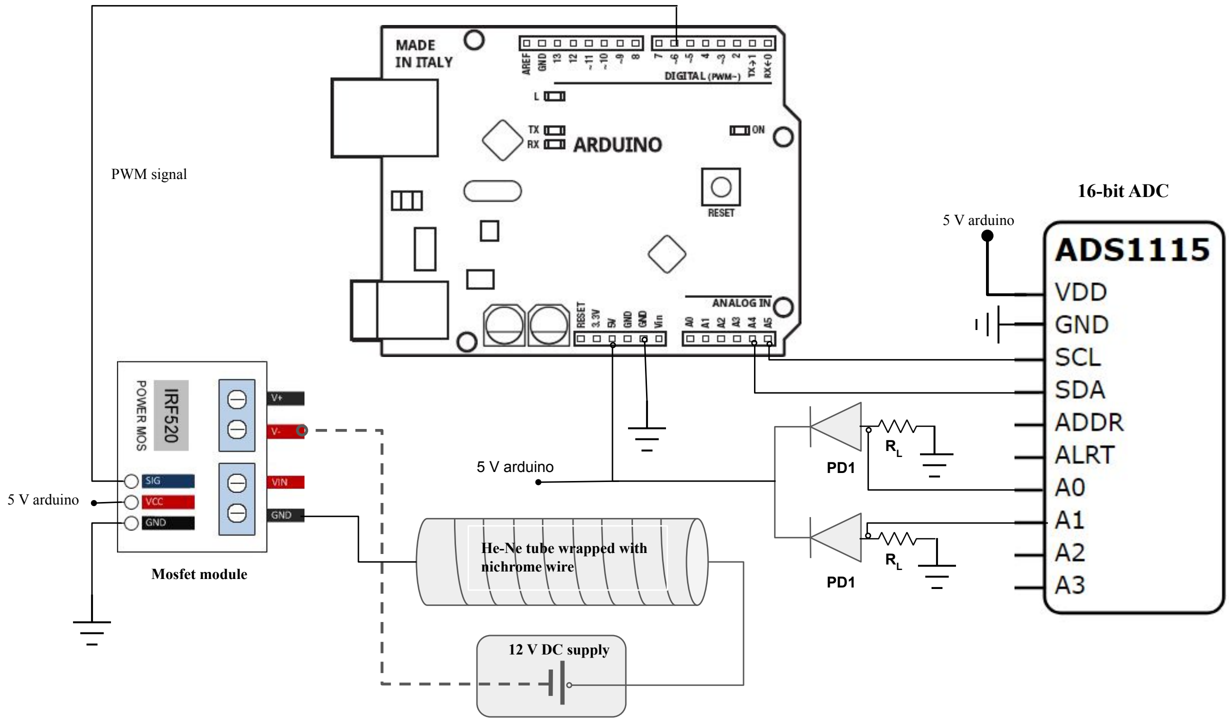

Figure 1: A schematic diagram of the curcuit used for feedback stabilization of He-Ne. PD1 and PD2 were denote the photodiodes and R_L is load resistance used (20 kOhm).

SI-2: PID code for microcontroller

Code - 1, For preheating the He-Ne laser cavity

```
//============================================//
// Preheating CODE for He-Ne laser cavity
//============================================//
int mosfet_sig = 6;  // the PWM signal goes to sig pin of mosfet module
int brightness_mosfet_led = 0;    // at zero time, led of mosfet off, its brightness value shows duty cycle of PWM signal
int increment_step = 5;    // step size to increase duty cycle for heating the he-ne laser tube

```

```
// the setup routine runs once when you press reset:
void setup() {
Serial.begin(9600);
// declare pin 9 to be an output:
pinMode(mosfet_sig, OUTPUT);
}

// the loop routine runs over and over again forever:
void loop() {
// set the brightness of pin 9:
analogWrite(mosfet_sig, brightness_mosfet_led);

// change the brightness for next time through the loop:
brightness_mosfet_led = brightness_mosfet_led + increment_step;

// reverse the direction if it reaches maximum duty cycle value
if (brightness_mosfet_led <= 0 ||brightness_mosfet_led >= 255) {
increment_step = -increment_step;
}
// wait for 30 milliseconds means that it will take almost 1.6 sec to reach maximum duty cycle value (100%)
delay(30);
}
```

Listing 1: Preheating CODE for He-Ne laser cavity

Code - 2, PID controller for He-Ne stablization

For our feedback control, we have used PID library version 1.2.1 (Brett Beauregard, https://github.com/br3ttb/Arduino-PID-Library). The variables used in this library are defined as given by Equation 1. In our case the error signal is defined as the difference of the signal of the normalized photo diode. For mode balance stabilization, the error signal must always be zero.

$$\text{output} = K_p e + K_d \frac{de}{dt} + K_i \int_0^t e(t)\, dt \tag{1}$$

Here, e = Setpoint - Input

```
//==========================================//
// PID CODE for HE-NE stabilization
//==========================================//
#include <PID_v1.h> //PID library
#include <Adafruit_ADS1X15.h>  //16 bit ADC library
Adafruit_ADS1115 ads;
float Voltage = 0.0;
float Voltage1 = 0.0;
const int numReadings =15; // averaging of photodiode signal to reduce noise
#define RELAY_PIN 6 // the PWM signal goes to sig pin of mosfet module (see Figure 1)
const int led = 6; //
double lightLevel;
int readings[numReadings];
int readings1[numReadings];// the readings from the analog input
int readIndex = 0;              // the index of the current reading
float total = 0;                  // the running total
float average = 0;                // the average
float total1 = 0;                  // the running total
float average1 = 0;                // the average

float norm=0;
float norm_pd1=0;
float norm_pd2=0;
float err_sig=0;

double Setpoint, Input, Output;  //These are just variables for storing values
PID myPID(&Input, &Output, &Setpoint,4.6, 0.050551851154,0.644488081098, DIRECT); // This sets up our PID Loop

const int sampleRate = 1; // Variable that determines how fast our PID loop runs12
// Communication setup
const long serialPing =180;
unsigned long now = 0; //This variable is used to keep track of time
// placehodler for current timestamp
unsigned long lastMessage = -10; //

void setup()
{
Serial.begin(9600);
```

```
// ============ seting the programmable gain amplifier (PGA) ==========================

//ads.setGain(GAIN_TWO);        +/- 2.048V  1 bit = 0.0625mV
//ads.setGain(GAIN_FOUR);      // +/- 1.024V  1 bit = 0.03125mV
//ads.setGain(GAIN_EIGHT);   //   +/- 0.512V  1 bit = 0.015625mV
ads.setGain(GAIN_SIXTEEN);    //+/- 0.256V  1 bit = 0.0078125mV
// ============ ============================================== ==========================

ads.begin();

Setpoint =0;
myPID.SetMode(AUTOMATIC);  //Turn on the PID loop
myPID.SetSampleTime(sampleRate); //Sets the sample rate
lastMessage = millis();
}

void loop()
{
int16_t adc0, adc1, adc2, adc3;
total = total - readings[readIndex];
total1 = total1 - readings1[readIndex];
// read from the sensor:
readings[readIndex] = ads.readADC_SingleEnded(0);
readings1[readIndex] = ads.readADC_SingleEnded(1);
// add the reading to the total:
total = total + readings[readIndex];
total1 = total1 + readings1[readIndex];

// advance to the next position in the array:
readIndex = readIndex + 1;

// if we're at the end of the array...
if (readIndex >= numReadings) {
// ...wrap around to the beginning:
readIndex = 0;
}

//====================== calculate the average value ==============================
average = total / numReadings;
average1 = total1 / numReadings;
Voltage =(average * 0.1875)/1000;
Voltage1 =(average1 * 0.1875)/1000;

norm = (Voltage+Voltage1);
norm_pd1=(Voltage/norm)*62000;
norm_pd2=(Voltage1/norm)*62000;
err_sig=(norm_pd2-norm_pd1);
//===============================================================================
int Setpoint =0; //Read our setpoint
lightLevel = err_sig;
Input = lightLevel; //Map it to the right scale
myPID.Compute();  //Run the PID loop
analogWrite(8, Output);

now = millis(); //Keep track of time
if(now - lastMessage > serialPing) {

//========================print all values==========================================

Serial.print( err_sig,5); // error signal that is difference of two normalized photodiodes signal
Serial.print(" ");

Serial.print(norm_pd1,5); // normalized signal at photodiode 1
Serial.print(" ");

Serial.print(norm_pd2,5); // normalized signal at photodiode 2
Serial.println(" ");
//===============================================================================
delay(.05);
lastMessage = now;
}}
```

Listing 2: PID code for He-Ne stabalization

SI-3: He-Ne laser cavity and Fizeau wedge characterization

In this section, some additional details of the calibration of the interferometer in order to establish a relation among fringe position (in pixels), frequency and FSR of the wedge.

Wedge spacing calibration with Dye laser

FSR of the wedge was estimated to be 20.2 GHz based on a measured physical thickness of 4.8 ± 0.1 mm (equation 2). Refractive index of UV fused silica (n_w) is 1.45717 at 632.8 nm and e_s is the wedge thickness. This FSR value is equal to the separation between two consecutive maxima or minima of interference pattern which is 129.30 pixels [see figure 2(c) in main manuscript].

$$FSR_w = \frac{c}{2\ n_w\ e_s} = 129.30 \ \text{ pixel} \tag{2}$$

For a more accurate measurement of FSR, we measured wedge spacing using a tunable dye laser. Our dye laser has minimum step size of 0.0001 nm and it is independently calibrated with iodine absorption spectrum. The changes in zeroth fringe position (ZFP) were measured by scanning the wavelength (with 0.002 nm step size) and is shown in figure 2 (left).

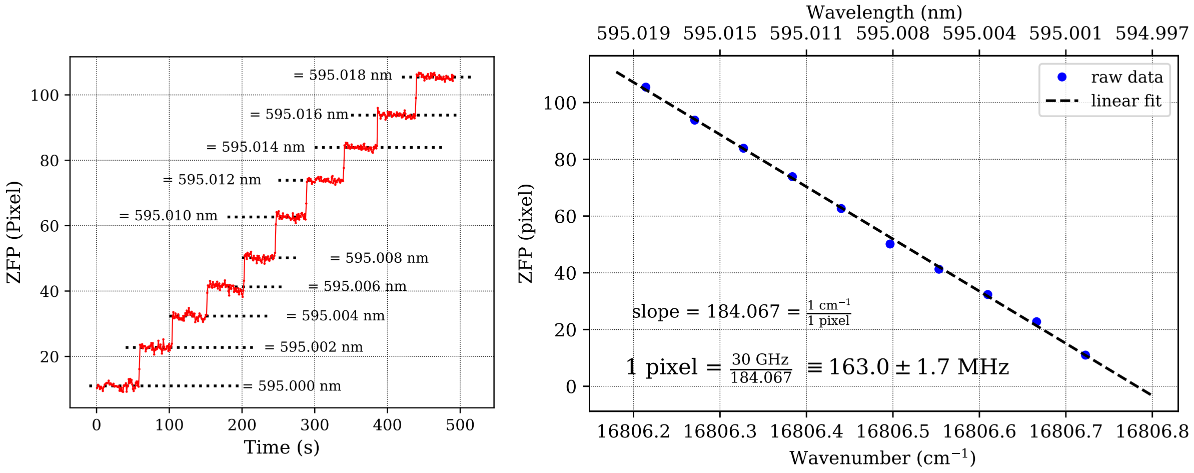

Figure 2: Left panel shows how Zeroth fringe position (ZFP) changes over wavelength with step size of 0.002 nm. In right panel, mean values of ZFP are plotted verses wavelength to get relation among ZFP shift (in pixel) and frequency.

Mean value of each measured ZFP distribution were plotted verses corresponding wavelength, as shown in figure 2 (right). From the slope of the linear fit we established a relation between change in ZPF (in pixel) and frequency. The FSR of fizeau wedge was calculated to be 21.1 ± 0.2 GHz (equation 2).

Determining FSR of the wedge using the longitudinal modes of He-Ne

The spacing between adjacent longitudinal modes of our He-Ne is 1078 MHz (as per data sheet).

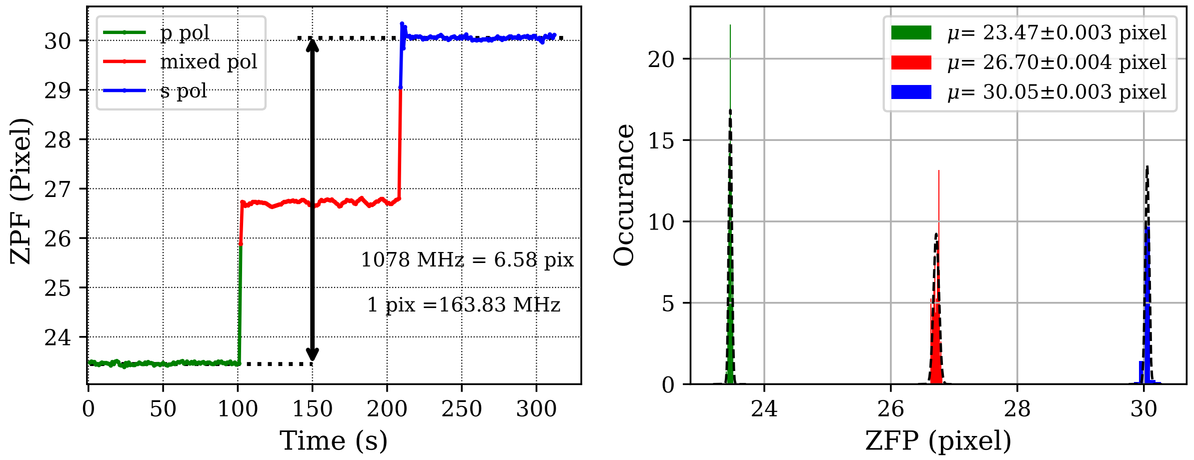

Figure 3: (Left) ZFP (in pixels) measured for single as well as mixed polarization using our interferometer.(right) Histogram plot of the ZFP measured for S, mixed polarization, and P polarization, respectively.

Using this fact we estimated the wedge spacing by monitoring the change in ZFP for adjacent modes (orthogonally polarized). We use a polarizing beam splitter cube (1000:1, discrimination) which allows select a single polarization to enter the interferometer at given time. Total change in ZFP for changing from S to P polarization was observed to be 6.58 pixel, as shown in figure 3 (left). Histogram of the measured ZPF distribution over time, for S, mixed and P polsarization, are shown in 3 (right).

Uncertainty in the mean value (standard error) of ZFP distributions for the S, mixed and P polarizations was measured to be 0.003, 0.004 and 0.003 pixels, respectively. The measured uncertainties showed that the interferometer had the ability to determine the change in ZPF better than 0.005 pixels. Having said that the thermal drift dominates at the longer time scales of 15 min (see manuscript figure 6). As a conservative estimate, we consider the interferometer drift of 0.02 pixel for 15 min as the overall uncertainty. Given that the frequency spacing between the adjacent modes is 1078 MHz, a relation among frequency shift and the fringe position deviation is readily obtained as 1 pixel = 163.83 MHz. Hence, FSR of the wedge was determined to be 21.183 ± 0.005 GHz.

Table 1: Calculated wedge spacing (mm) and its FSR (GHz) from estimated using different methods

Method	Calibration reference	wedge spacing (mm)	FSR of wedge (GHz)
Physical thickness measurement	0.1 mm least count vernier caliper	4.8	21.4 ± 0.4
Tunable Dye laser	Iodine absorption spectrum	4.87	21.1 ± 0.2
Adjacent He-Ne modes	FSR of the He-Ne cavity	4.857	21.183 ± 0.005

All these methods give results consistent with each other. Measuremnts with a tunable dye laser and adjacent He-Ne modes provide successively higher accuracy and precision.

SI-4: Mode hop free scanning under Neon gain profile

In general, He-Ne laser is used as a single wavelength laser. However, the feedback system implemented by us allows us to tune its wavelength in a controlled manner. To verify tuning and mode hop scanning, we were measured the fringe pattern from single polarization (using polarizing beam splitter cube) in the interferometer. The ZFP was measured for a passively controlled cavity, and mode hop free scan was observed, as indicated by the fringe position shift (figure 4). This can also be done actively using the feedback system with a variable setpoint to obtain an electronically controlled scan.

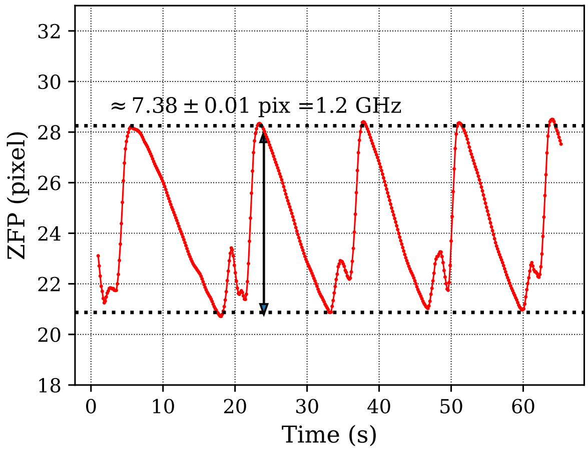

Figure 4: ZFP verses time for a single polarization of He-Ne. In these measurements active feedback was turned off and the cavity was slowly allowed to drift (thermally).

A total tuning was 1.2 GHz observed, which is consistent with the expected gain bandwidth of the He–Ne laser.

SI-5: Effect of back reflection on locking stability

We quantified this disturbance by measuring the error signal with and without fiber coupling which is shown in figure 5. We found that the stability of our He-Ne laser is almost 2-3 times poor when it is coupled using the fiber causing a small portion of the back reflected light entering the He-Ne cavity. This could be avoided by adjusting the alignment such that there is a slight angular offset or using free space coupling.

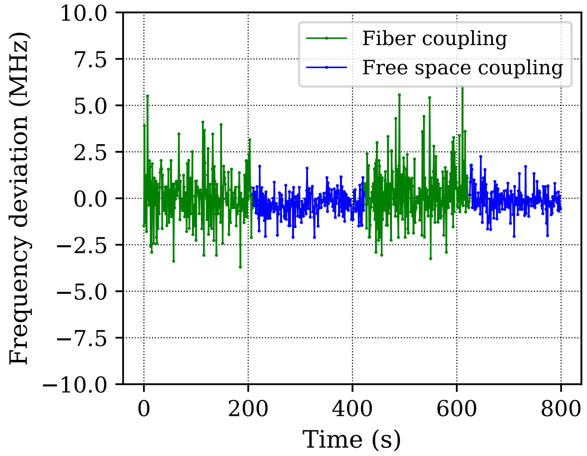

Figure 5: Frequency drift observed with and without fiber coupling. Frequency instability was observed to increase by a factor of 2-3 (caused back reflection). These measurements were made with the 10-bit ADC.
